# Age-stratified safety signal mining of ADHD medications in pediatric populations using the FAERS database (2014–2024): a pharmacovigilance study using multi-method disproportionality analysis

**DOI:** 10.3389/fped.2026.1865791

**Published:** 2026-09-16

**Authors:** Guangping Qin, Ling Chen

**Affiliations:** Department of Pediatrics, Pingxiang Municipal People's Hospital, Pingxiang, Jiangxi, China

**Keywords:** ADHD, disproportionality analysis, FAERS, pediatrics, pharmacovigilance

## Abstract

**Background:**

Pediatric attention-deficit/hyperactivity disorder (ADHD) medications have been analyzed in the FDA Adverse Event Reporting System (FAERS), yet systematic age-stratified signal profiling under ICH E11(R1) developmental categories, covering the recently approved agents viloxazine and serdexmethylphenidate, remains unreported.

**Methods:**

We performed a retrospective disproportionality analysis of pediatric (age <18 years) FAERS reports, 2014Q1–2024Q4, after FDA-standard three-step deduplication. Ten active ingredients (eight in the main analysis, two in a new-drug branch) were identified. Reporting odds ratios (ROR), proportional reporting ratios (PRR), information components (IC, Bate 1998 uniform prior), and empirical Bayesian geometric means (EBGM, DuMouchel 1999) were computed. A signal was classified as Strong when positive across all four methods. Seven prespecified sensitivity analyses were conducted.

**Results:**

Among 15,301 pediatric ADHD cases, 100 Strong signals were identified (71 in adolescents, 29 in children). *α*2-agonists accounted for 79/100 (clonidine-ER 52, guanfacine-ER 27). A novel dextroamphetamine–posterior reversible encephalopathy syndrome (PRES) signal was reproducible across three independent sensitivity analyses. Methylphenidate showed no Strong signals under within-class or full-pediatric comparators.

**Conclusion:**

Distinct age-specific safety signals emerged for ADHD medications, with dextroamphetamine–PRES warranting targeted pharmacovigilance attention.

## Introduction

Attention-deficit/hyperactivity disorder (ADHD) affects 5%–7% of children and adolescents worldwide and remains among the most commonly treated neurodevelopmental conditions in pediatric practice (1, 2). Pharmacotherapy is a cornerstone of management for moderate-to-severe cases, with the US Food and Drug Administration (FDA) approving a range of psychostimulants (including methylphenidate, dexmethylphenidate, amphetamine formulations, dextroamphetamine, lisdexamfetamine) and non-stimulants (atomoxetine, guanfacine extended-release, clonidine extended-release) for pediatric use (3). The recent approvals of viloxazine (Qelbree, 2021) and the serdexmethylphenidate-containing product Azstarys (2021) have further broadened the therapeutic armamentarium. Despite decades of use, concerns regarding rare but serious adverse events—particularly in developmentally distinct subpopulations—continue to motivate post-marketing surveillance efforts.

Randomized controlled trials (RCTs), while essential for establishing efficacy, are statistically underpowered to detect rare adverse events occurring at rates below 1/1,000 and typically enroll children over narrow age ranges with limited follow-up. The FDA Adverse Event Reporting System (FAERS) provides a complementary data source for detecting such rare signals across real-world use, and disproportionality analysis (DA)—including the reporting odds ratio (ROR), proportional reporting ratio (PRR), information component (IC) from the Bayesian confidence propagation neural network (BCPNN), and the empirical Bayesian geometric mean (EBGM) from the multi-item gamma Poisson shrinker (MGPS)—has emerged as the methodological standard for hypothesis-generating pharmacovigilance (4–6). The 2024 READUS-PV reporting guideline has codified best practices for such analyses (7).

Prior FAERS investigations of ADHD medications, however, have several limitations that the present study aims to address. First, most analyses either pool pediatric and adult reports or use coarse binary age stratification (<18 vs. ≥18 years), rather than the developmentally distinct strata recommended by ICH E11(R1) (infants and toddlers, children, adolescents) (8). Second, systematic cross-comparisons across all current FDA-approved pediatric ADHD agents have rarely included the recently approved viloxazine and serdexmethylphenidate. Third, full methodological transparency—including prespecified signal thresholds, MedDRA version declaration, sparse-cell handling, and open-source code—has been inconsistent. Fourth, Wu and colleagues (2025) analyzed only three ADHD agents (methylphenidate, atomoxetine, amphetamine) without age stratification (9), and Wei and colleagues (2023) covered 2014–2022 with age grouping but limited drug coverage (10). To our knowledge, no prior study has integrated (i) comprehensive coverage of current pediatric ADHD agents, (ii) ICH E11(R1) age stratification, (iii) four-method combined DA with prespecified signal classification, and (iv) READUS-PV–compliant reporting in a single pharmacovigilance study.

The aim of this study was to systematically profile age-specific safety signals of ADHD medications in pediatric populations using FAERS data from 2014 to 2024, applying four prespecified disproportionality methods with rigorous signal classification and seven sensitivity analyses, to identify both established and novel drug–event associations that warrant further pharmacovigilance attention. A contemporaneous pharmacovigilance comparison of viloxazine and dextroamphetamine across all age groups has recently appeared in the VigiAccess/FAERS literature (11), but without pediatric age stratification or four-method concordance classification; the present study extends that work into a developmentally-stratified and methodologically-integrated framework.

## Methods

### Data source

Quarterly ASCII data files from the FDA Adverse Event Reporting System (FAERS) for 2014Q1 through 2024Q4 (44 consecutive quarters) were downloaded from the FDA open data portal (accessed April 2026) (12). FAERS is a passive surveillance database containing spontaneous reports of adverse drug events submitted by healthcare professionals, consumers, and manufacturers worldwide. The database comprises seven interlinked tables: DEMO (demographics), DRUG (drug exposures and role codes), REAC (adverse reactions), OUTC (outcomes), INDI (drug indications), THER (therapy dates), and RPSR (report sources). Adverse-event terms were analyzed at the MedDRA Preferred Term (PT) level using the PT values supplied with each FAERS quarterly release, which are coded under the MedDRA version current at the time of each report. Because an MSSO MedDRA subscription was not available, no systematic cross-version remapping was performed; PTs renamed across versions are retained under their most recent FAERS value. The reference version for interpretation is MedDRA v27.0. The extent of potential terminology drift across the 2014–2024 window is documented in Supplementary Table S2 and addressed in the Limitations. The analytic window (2014Q1–2024Q4) was pre-specified and version-locked prior to data extraction; although the database was accessed in April 2026, quarters after 2024Q4 were deliberately excluded because the most recent FAERS quarters remain subject to ongoing case revision and characteristic reporting lag. The consequently limited post-marketing accrual for the two 2021-approved agents is analyzed in the new-drug branch (sensitivity analysis S4) and acknowledged in the Limitations.

### Study design

This was a retrospective case/non-case disproportionality analysis with ICH E11(R1)-aligned age stratification. The study protocol was predeclared prior to data analysis, with all signal thresholds, age cutoffs, drug dictionary, and sensitivity analyses fixed in a version-controlled project document; all subsequent deviations were logged in a project deviation ledger. While this analysis was not formally registered on a pre-registration platform (e.g., OSF), the complete analysis protocol—including the drug dictionary, signal thresholds, and sensitivity-analysis battery—was version-locked in a project repository prior to data extraction, with git commit timestamps establishing temporal priority; the full repository will be deposited on Zenodo upon publication. The study adhered to the READUS-PV 2024 reporting guideline (7).

### Case identification and deduplication

Following the FDA-recommended three-step deduplication procedure outlined in the FAERS quarterly data documentation, for each unique CASEID we retained the record with (i) the latest FDA_DT, (ii) the largest PRIMARYID in case of FDA_DT ties, and (iii) excluded all reports appearing in FDA's quarterly nullified-case lists (available from 2019Q1 onward). For the purpose of time-trend analyses, INIT_FDA_DT (first-received date) was used rather than FDA_DT (latest-revised date) to avoid reverse time contamination.

### Age standardization and stratification

Patient age was computed by combining the AGE and AGE_COD fields in DEMO using the conversion factors [1 year for YR; 1/12 for MON; 1/52 for WK; 1/365.25 for DY; 10 for DEC; 1/(365.25 × 24) for HR]. Values <0 or >125 were set to missing, as were records with uncoded age units. Pediatric reports were defined as age_years <18. Age groups were assigned following ICH E11(R1) developmental categories: infants and toddlers (28 days to <2 years), children (2 to <12 years), and adolescents (12 to <18 years) (8). An adaptive rule was prespecified: the infants-and-toddlers stratum would be analyzed as an independent main stratum only if *n* ≥ 500, otherwise relegated to a sensitivity descriptive case series.

### Drug identification

A curated dictionary of 10 active ingredients was assembled based on current FDA labeling: six stimulants (methylphenidate, dexmethylphenidate, amphetamine including mixed amphetamine salts, dextroamphetamine, lisdexamfetamine, serdexmethylphenidate) and four non-stimulants (atomoxetine, viloxazine, guanfacine extended-release, clonidine extended-release). Two additional FDA-approved stimulants (methamphetamine and levoamphetamine) were excluded *a priori* because methamphetamine is essentially unused in pediatric ADHD practice and levoamphetamine is a component of mixed amphetamine salt preparations rather than an independently approved product. Drug–ingredient matching was performed on both DRUGNAME and PROD_AI fields using a case-insensitive regular-expression dictionary covering brand names, spelling variants, and combination formulations. Serdexmethylphenidate and viloxazine, both approved in 2021, were prespecified as a separate “new-drug branch” with analyses restricted to 2021Q2–2024Q4 against year-matched comparators, to minimize the Weber effect on disproportionality estimates.

### Variable definitions

The primary exposure was defined as active ingredient (INN-level) for role_cod = “PS” (primary suspect) in the main analysis; sensitivity analyses relaxed this to include secondary suspect (“SS”). The primary outcome was the occurrence of a specific MedDRA preferred term (PT) in REAC, with SOC- and HLT-level summaries as secondary outcomes. Indication (from INDI.INDI_PT) was used as a confounder and also as the basis for a sensitivity subset restricted to ADHD-related indications. Serious outcome flags followed the ICH E2B definition (any of death, life-threatening, hospitalization, disability, or congenital anomaly). Reporter country (DEMO.REPORTER_COUNTRY) and reporter type (DEMO.OCCP_COD) were retained as descriptive covariates. Concomitant drug count was computed as the number of distinct active ingredients per report minus one, after INN-level aggregation of multiple formulations of the same drug.

### Disproportionality analysis

For each (INN, age_group, MedDRA PT) combination with ≥3 observed cases, a 2 × 2 contingency table was constructed using the within-class pediatric ADHD comparator (reports of the other seven main-analysis INNs within the same age_group as non-cases). Four signal measures were computed using prespecified formulas (13): ROR = (a × d)/(b × c) with log-normal 95% confidence interval (CI) (13); PRR = [a/(a + b)]/[c/(c + d)] with Yates-corrected chi-square (14); IC (BCPNN) = log₂[E(P(D,R))/(E(P(D)) × E(P(R)))] using the Bate 1998 uniform prior (*α* = *β* = *γ* = 1); IC_025_ was computed as IC—1.96 × √Var(IC) (15); and EBGM (MGPS) from the gamma–Poisson shrinker with the DuMouchel 1999 empirical Bayes procedure; EBGM_05_ denotes the fifth percentile of the posterior distribution (16).

Here, cell a denotes the number of reports of the index INN with the index preferred term; b, reports of the index INN with all other preferred terms; c, reports of the comparator INNs (the other seven main-analysis ingredients in the same age group) with the index preferred term; and d, reports of the comparator INNs with all other preferred terms, as summarized below.

**Table:** 

	Event of interest (index PT)	All other events
Index INN	a	b
Comparator INNs	c	d

For sparse cells with a <5, Mid-P exact confidence intervals were used for ROR and PRR in place of the log-normal approximation. Prespecified signal thresholds were: ROR 95% CI lower bound >1; PRR ≥2 with *χ*^2^ ≥ 4; IC_025_ > 0; and EBGM_05_ ≥ 2 (4, 7). A combined signal classification was then applied: Strong (positive across all four methods), Moderate (3/4), Weak (2/4), and None (≤1/4). Only Strong signals were reported in the main text; Moderate and Weak signals were reserved for supplementary reporting. Consistent with standard practice in pharmacovigilance disproportionality analysis and the READUS-PV 2024 guideline (7, 17), no family-wise multiplicity correction (e.g., Bonferroni or Benjamini-Hochberg false discovery rate) was applied across the 2,679 tested drug-event combinations; instead, signal robustness was established through the conjunctive four-method concordance criterion and seven prespecified sensitivity analyses.

### Sensitivity analyses

Seven prespecified sensitivity analyses were conducted to assess signal robustness: S1, relaxation of role_cod from “PS” to “PS + SS”; S2, full pediatric FAERS comparator (entire pediatric FAERS pool rather than within-class); S3, active-comparator–restricted disproportionality analysis with methylphenidate as reference drug; S4, new-drug branch disproportionality analysis (viloxazine and serdexmethylphenidate against year-matched 2021Q2–2024Q4 controls); S5, descriptive case series of the infants-and-toddlers stratum; S6, serious-outcome subset per ICH E2B criteria; S7, marketing era stratification (2014–2017, 2018–2020, 2021–2024). An originally planned interrupted time series of the 2006 FDA black-box warning was removed because it fell outside the study time window.

### Missing data handling

Age-missing records were excluded from main analyses and their distribution patterns reported descriptively. Other fields with missingness (reporter type, reporter country) were retained as explicit “Unknown” categories. Multiple imputation was not performed, given the missing-not-at-random nature typical of spontaneous reports.

### Software and reproducibility

Data processing and ROR/PRR/IC computations were performed in Python 3.14 (pandas 2.3.3, SciPy 1.17.1, NumPy 2.4.3, pyarrow 23.0.1). EBGM computations used R 4.5.3 with the openEBGM 0.9.1 package (18); Python and R interoperated via subprocess calls with intermediate CSV exchange. Python implementations of ROR, PRR, and IC were validated against three literature-derived 2 × 2 ground-truth fixtures [haloperidol–neuroleptic malignant syndrome from van Puijenbroek 2002 (13); cerivastatin–rhabdomyolysis from Evans 2001 (14); captopril–cough from Bate 1998 (15)] with all measures agreeing within ±1% of published values, and independently cross-validated against the scipy.stats.chi2_contingency implementation.

### Ethical considerations

FAERS is a publicly accessible database of de-identified spontaneous adverse event reports. This study used only anonymized, publicly available data and did not involve direct human-subject research or identifiable private information; therefore, institutional review board approval and informed consent were not required.

## Results

### Study population and baseline characteristics

The deduplicated FAERS database for 2014Q1–2024Q4 contained 15,691,791 unique cases, of which 14,695,692 fell within the study time window. After applying pediatric (age <18 years) and drug-matching inclusion criteria with role_cod = “PS”, the final main analysis set comprised **15,301 unique pediatric ADHD cases** (Figure 1). Stratified by ICH E11(R1) age categories, 296 cases (1.9%) were infants and toddlers, 9,184 (60.0%) were children, and 5,821 (38.0%) were adolescents. Per the prespecified adaptive rule, the infants-and-toddlers stratum was relegated to descriptive sensitivity analysis S5.

**Figure 1:**
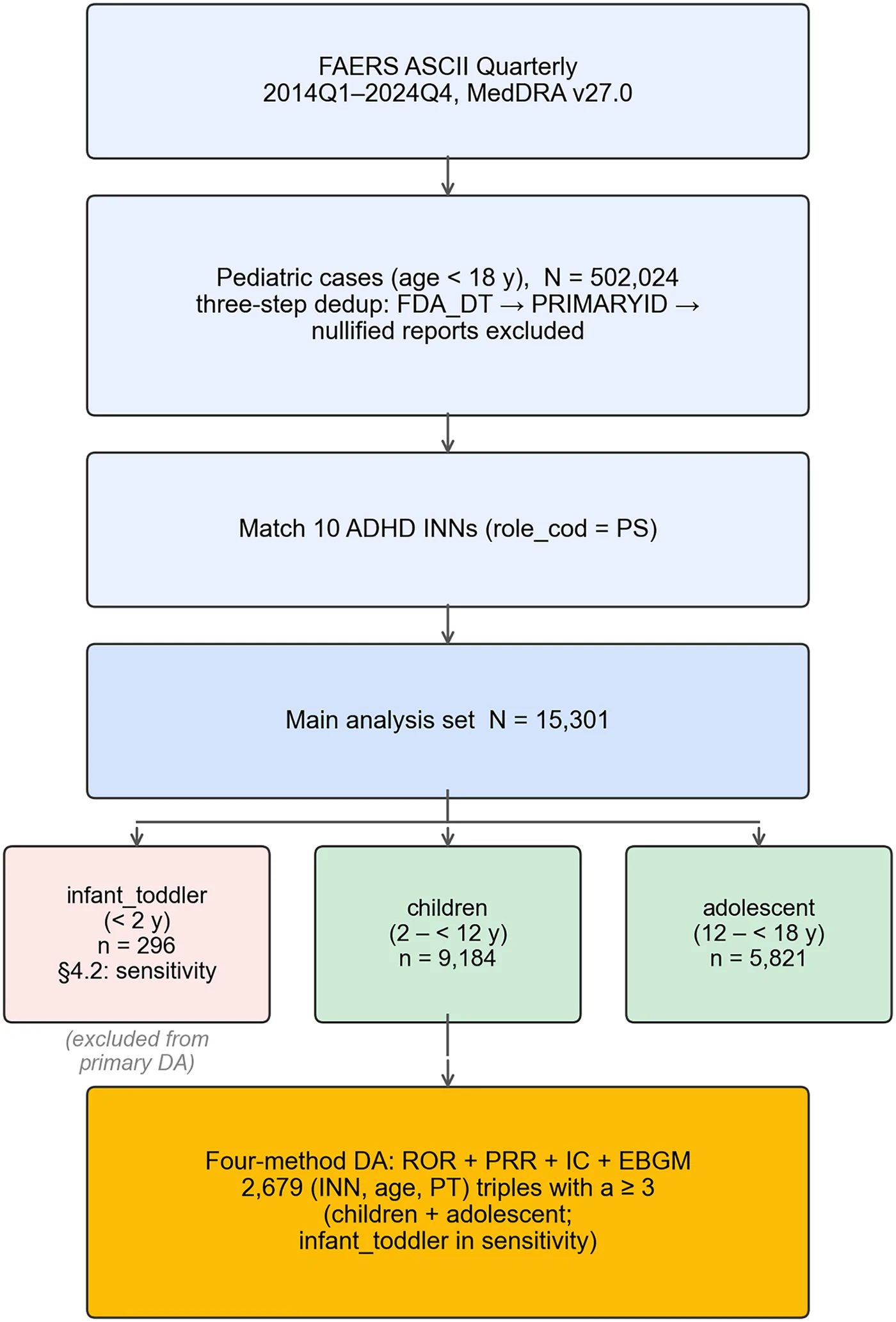
STROBE flow diagram of pediatric ADHD FAERS main-analysis set construction. Case selection from the FAERS ASCII Quarterly database (2014Q1–2024Q4, MedDRA v27.0) through three-step deduplication (FDA_DT → PRIMARYID → exclusion of nullified reports), pediatric restriction (age <18 years; *N* = 502,024), ADHD drug matching (ten International Nonproprietary Names, role_cod = PS), and age stratification under the ICH E11(R1) developmental framework. The main analysis set (*N* = 15,301) comprises children (2 to <12 years, *n* = 9,184) and adolescents (12 to <18 years, *n* = 5,821); the infant-and-toddler stratum (*n* = 296) was relegated to sensitivity analysis S5 per the prespecified adaptive rule. The primary four-method disproportionality analysis (ROR + PRR + IC + EBGM) was performed on 2,679 (INN, age, PT) triples satisfying a ≥ 3.

Males predominated across all age groups (overall 62.6%; children 64.9%; adolescents 59.6%), consistent with the established male predominance of pediatric ADHD diagnosis (19). The United States contributed 74.4% of all reports. Consumer-initiated reports accounted for 53.2% of cases—more than triple the proportion reported by physicians (17.6%), a reporting profile substantially biased toward parent/caregiver-driven submissions and distinct from classical pharmacovigilance data dominated by healthcare professionals. Stimulant-class agents represented 73.7% of exposures overall, though this proportion fell to 49.3% in infants-and-toddlers, where non-stimulants (primarily clonidine-ER) dominated. Of clinical and methodological note, only 49.3% of cases had ADHD explicitly recorded as the indication, while 43.8% were coded with mixed or non-ADHD indications, reflecting the widespread off-label use of these agents. The reporting era was skewed toward earlier years (2014–2017: 57.2%), with a notable increase in the 2021–2024 era (24.0%) coinciding with COVID-19–related expansion of telepsychiatric ADHD diagnosis and the approval of viloxazine and serdexmethylphenidate.

A striking differential emerged in outcome severity across age strata: serious adverse events (per ICH E2B criteria) occurred in 67.2% of infants-and-toddlers reports, compared with only 12.5% in children and 23.2% in adolescents—a > 5-fold enrichment suggesting that the handful of reports involving very young children disproportionately represent severe, possibly off-label or exposure-during-pregnancy events (corroborated by the top preferred term in S5 being “Foetal exposure during pregnancy”). Full distributional details are provided in Table 1.

**Table 1:** Baseline characteristics of pediatric ADHD reports (FAERS 2014–2024).

Characteristic	Infants & toddlers (*n* = 296)	Children (*n* = 9,184)	Adolescents (*n* = 5,821)	Overall (*n* = 15,301)
Sex, *n* (%)				
Male	150 (50.7)	5,961 (64.9)	3,470 (59.6)	9,581 (62.6)
Female	116 (39.2)	1,963 (21.4)	1,835 (31.5)	3,914 (25.6)
Unknown	30 (10.1)	1,260 (13.7)	516 (8.9)	1,806 (11.8)
Reporter country, *n* (%)				
United States	175 (59.1)	7,265 (79.1)	3,948 (67.8)	11,388 (74.4)
Japan	6 (2.0)	238 (2.6)	260 (4.5)	504 (3.3)
Canada	21 (7.1)	257 (2.8)	181 (3.1)	459 (3.0)
Sweden	0 (0.0)	55 (0.6)	308 (5.3)	363 (2.4)
United Kingdom	6 (2.0)	151 (1.6)	163 (2.8)	320 (2.1)
Other	88 (29.7)	1,218 (13.3)	961 (16.5)	2,267 (14.8)
Reporter type, *n* (%)				
Consumer	63 (21.3)	5,202 (56.6)	2,868 (49.3)	8,133 (53.2)
Physician	81 (27.4)	1,505 (16.4)	1,100 (18.9)	2,686 (17.6)
Other health professional	66 (22.3)	586 (6.4)	692 (11.9)	1,344 (8.8)
Pharmacist	10 (3.4)	676 (7.4)	383 (6.6)	1,069 (7.0)
Other/Unknown	76 (25.7)	1,215 (13.2)	778 (13.4)	2,069 (13.5)
Drug class, *n* (%)				
Stimulant	146 (49.3)	6,825 (74.3)	4,302 (73.9)	11,273 (73.7)
Non-stimulant	150 (50.7)	2,359 (25.7)	1,519 (26.1)	4,028 (26.3)
Indication, *n* (%)				
ADHD only	30 (10.1)	4,862 (52.9)	2,655 (45.6)	7,547 (49.3)
Mixed or non-ADHD	236 (79.7)	3,664 (39.9)	2,800 (48.1)	6,700 (43.8)
No indication recorded	30 (10.1)	658 (7.2)	366 (6.3)	1,054 (6.9)
Concomitant drugs, median (IQR)	2 (1–4)	1 (1–2)	2 (1–3)	1 (1–3)
Outcome severity, *n* (%)				
Serious (ICH E2B)	199 (67.2)	1,145 (12.5)	1,349 (23.2)	2,693 (17.6)
Non-serious	68 (23.0)	2,278 (24.8)	1,650 (28.3)	3,996 (26.1)
Outcome not recorded	29 (9.8)	5,761 (62.7)	2,822 (48.5)	8,612 (56.3)
Reporting era, *n* (%)				
2014–2017	74 (25.0)	5,624 (61.2)	3,052 (52.4)	8,750 (57.2)
2018–2020	98 (33.1)	1,662 (18.1)	1,112 (19.1)	2,872 (18.8)
2021–2024	124 (41.9)	1,898 (20.7)	1,657 (28.5)	3,679 (24.0)

Percentages within each age group sum to 100% for each characteristic. ICH E2B serious criteria: death, life-threatening, hospitalization, disability, or congenital anomaly.

IQR, interquartile range.

### Main disproportionality analysis: 100 strong signals

Across 2,679 drug–event combinations meeting the minimum case count (a ≥ 3), 100 were classified as Strong signals (four-method concordance), 488 as Moderate, 149 as Weak, and 1,942 as None (Figure 1A). The prespecified Mid-P exact confidence interval procedure was applied to 969 sparse cells (36.2% of combinations), ensuring valid inference under low cell counts.

Strong signals were disproportionately concentrated in α2-adrenergic agonists: clonidine-ER accounted for 52 Strong signals (52%) and guanfacine-ER for 27 (27%), together comprising 79 of the 100 Strong signals. Stimulants contributed fewer signals overall — dextroamphetamine (8 Strong), amphetamine (5), lisdexamfetamine (4); among other non-stimulants, atomoxetine contributed 4 Strong signals. Notably, methylphenidate alone, accounting for 7,856 (51.3%) of the main analysis set, showed zero Strong signals under within-class comparison — a finding robust to the full pediatric FAERS comparator (sensitivity analysis S2).

Strong signals exhibited marked age-group asymmetry: 71 of 100 (71%) occurred in adolescents vs. 29 (29%) in children (Table 2), disproportionate to their respective case counts (adolescents 38%, children 60% of main set; ratio of Strong signal prevalence per case: 0.0122 vs. 0.0032, *p* < 0.001 by *χ*^2^). This enrichment persisted across all sensitivity analyses.

**Table 2:** Top 10 strong signals in the main analysis (ranked by IC_025_).

#	Age group	Drug	Preferred term	a	ROR (95% CI)	PRR	IC (IC_025_)	EBGM (EBGM_05_)	CI method	In consensus set (main ∩ S1 ∩ S6)
1	Adolescent	Clonidine-ER	Obesity	19	516.0 (68.7–3,873.5)	472.6	4.62 (3.72)	21.4 (14.4)	log-normal	No
2	Children	Clonidine-ER	Haematuria	31	115.7 (48.0–279.0)	107.3	4.19 (3.50)	16.3 (12.0)	log-normal	No
3	Adolescent	Clonidine-ER	Overweight	14	185.6 (41.9–821.8)	174.1	4.51 (3.49)	19.1 (12.0)	log-normal	No
4	Adolescent	Clonidine-ER	Blood prolactin increased	15	133.2 (38.3–463.5)	124.4	4.44 (3.46)	18.5 (11.8)	log-normal	No
5	Children	Clonidine-ER	Miosis	25	91.9 (37.5–225.3)	86.5	4.14 (3.38)	15.4 (10.9)	log-normal	Yes
6	Children	Clonidine-ER	Bradycardia	72	46.0 (30.7–68.9)	38.3	3.82 (3.38)	13.6 (11.1)	log-normal	Yes
7	Adolescent	Clonidine-ER	Mental status changes	17	76.1 (29.7–195.1)	70.5	4.27 (3.37)	16.9 (11.1)	log-normal	Yes
8	Children	Clonidine-ER	Hypotension	71	45.2 (30.2–67.8)	37.8	3.82 (3.37)	13.5 (11.1)	log-normal	Yes
9	Adolescent	Dextroamphetamine	Metabolic alkalosis	4	161.0 (20.0–3,983.3)	156.6	5.05 (3.33)	16.5 (6.6)	Mid-P	No
10	Adolescent	Clonidine-ER	Miosis	9	233.1 (29.4–1,848.4)	223.8	4.55 (3.30)	17.9 (10.0)	log-normal	Yes

ROR, reporting odds ratio; PRR, proportional reporting ratio; IC, information component (Bayesian); IC_025_, lower 2.5 percentile of IC; EBGM, empirical Bayesian geometric mean; EBGM_05_, 5th percentile of EBGM posterior; CI, confidence interval.

### Age-specific signal patterns by active ingredient

Figure 2A presents a heatmap of IC₀₂₅ values across all eight main-analysis ingredients stratified by age group for the top 10 MedDRA preferred terms by signal count. The clonidine-ER and guanfacine-ER columns showed consistently elevated signals across cardiovascular (bradycardia, hypotension), ophthalmic (miosis), endocrine (prolactin elevation), and metabolic PTs. In contrast, methylphenidate and dexmethylphenidate signals were uniformly attenuated or inverse, consistent with their role as the dominant reference drugs in the within-class comparator pool. Annual Strong-signal counts for the three top-producing ingredients are shown in Figure 2B; temporal stability is evident across the study period, arguing against any single-year reporting spike as an explanation for the cumulative signal concentrations.

**Figure 2:**
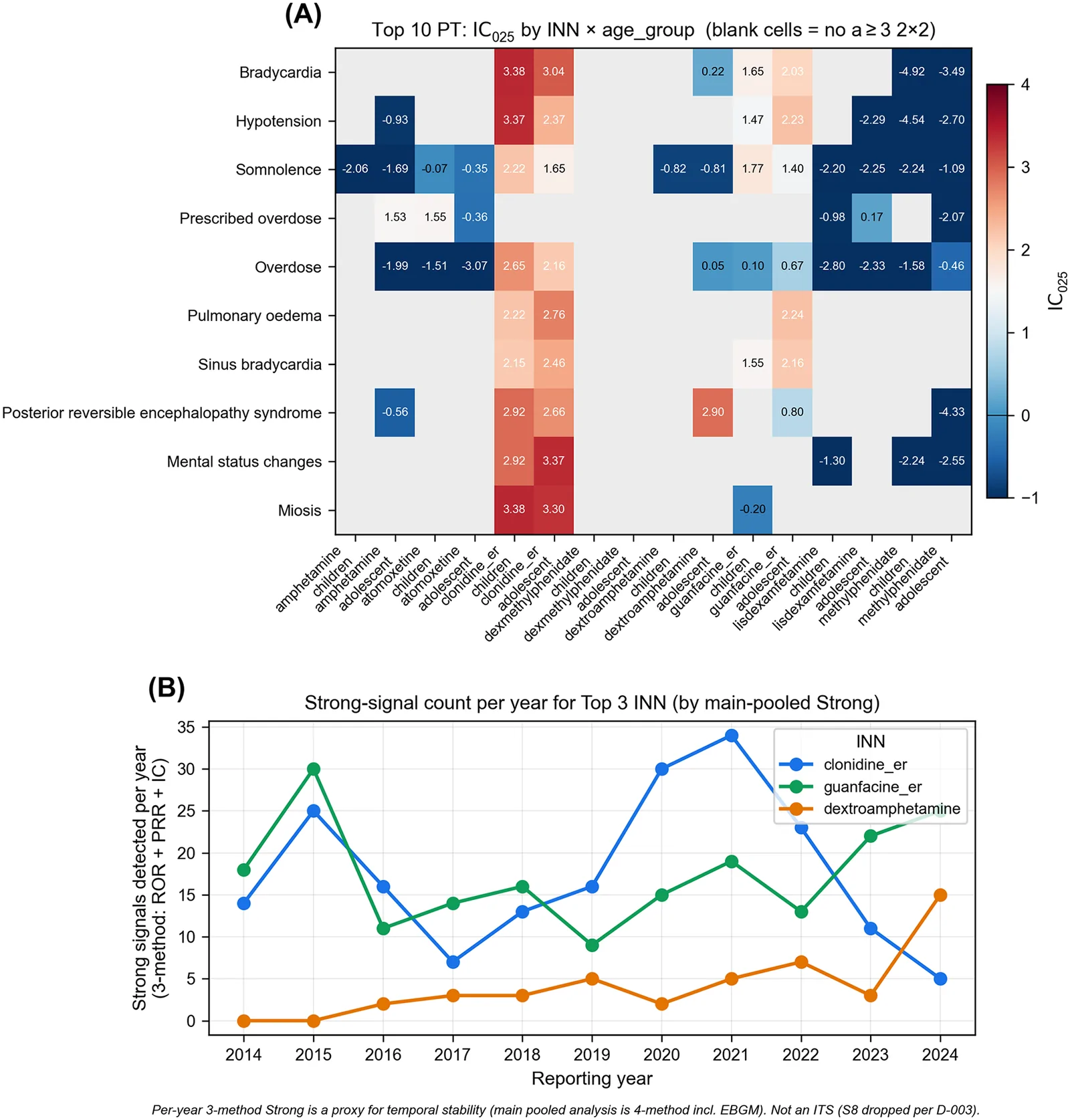
Age-specific signal intensity **(A)** and annual Strong-signal trajectory **(B)**, 2014–2024. **(A)** Heatmap of Information Component lower 2.5-percentile (IC_025_) across the top 10 MedDRA preferred terms by signal count, stratified by International Nonproprietary Name × age group. Warm colors denote positive disproportionality (IC_025_ > 0); cool colors denote inverse disproportionality; light-grey cells indicate (INN, age, PT) triples not meeting the minimum case-count threshold (a < 3). Numerical values within cells are IC_025_ point estimates. **(B)** Annual signal counts for the three top-ranking International Nonproprietary Names by pooled Strong-signal count (clonidine-ER, guanfacine-ER, dextroamphetamine). For a stable year-by-year comparison, panel B applies a three-method concordance criterion (ROR + PRR + IC all positive); this is deliberately distinct from the four-method “Strong” classification used for the main analysis (which additionally requires EBGM_05_ ≥ 2), because the empirical Bayesian EBGM cannot be stably estimated within single-year strata of low case count. Panel B is therefore a descriptive indicator of temporal stability and should not be interpreted as a count of four-method Strong signals or as an interrupted time-series estimate.

Individual drug–event associations were further examined via age-stratified forest plots (Figure 3). For several signals, confidence intervals overlapped between children and adolescent strata (e.g., clonidine-ER–bradycardia: children ROR 46.0), suggesting class-wide effects. For others, age-specific enrichment was evident (e.g., dextroamphetamine–metabolic alkalosis: adolescents only).

**Figure 3:**
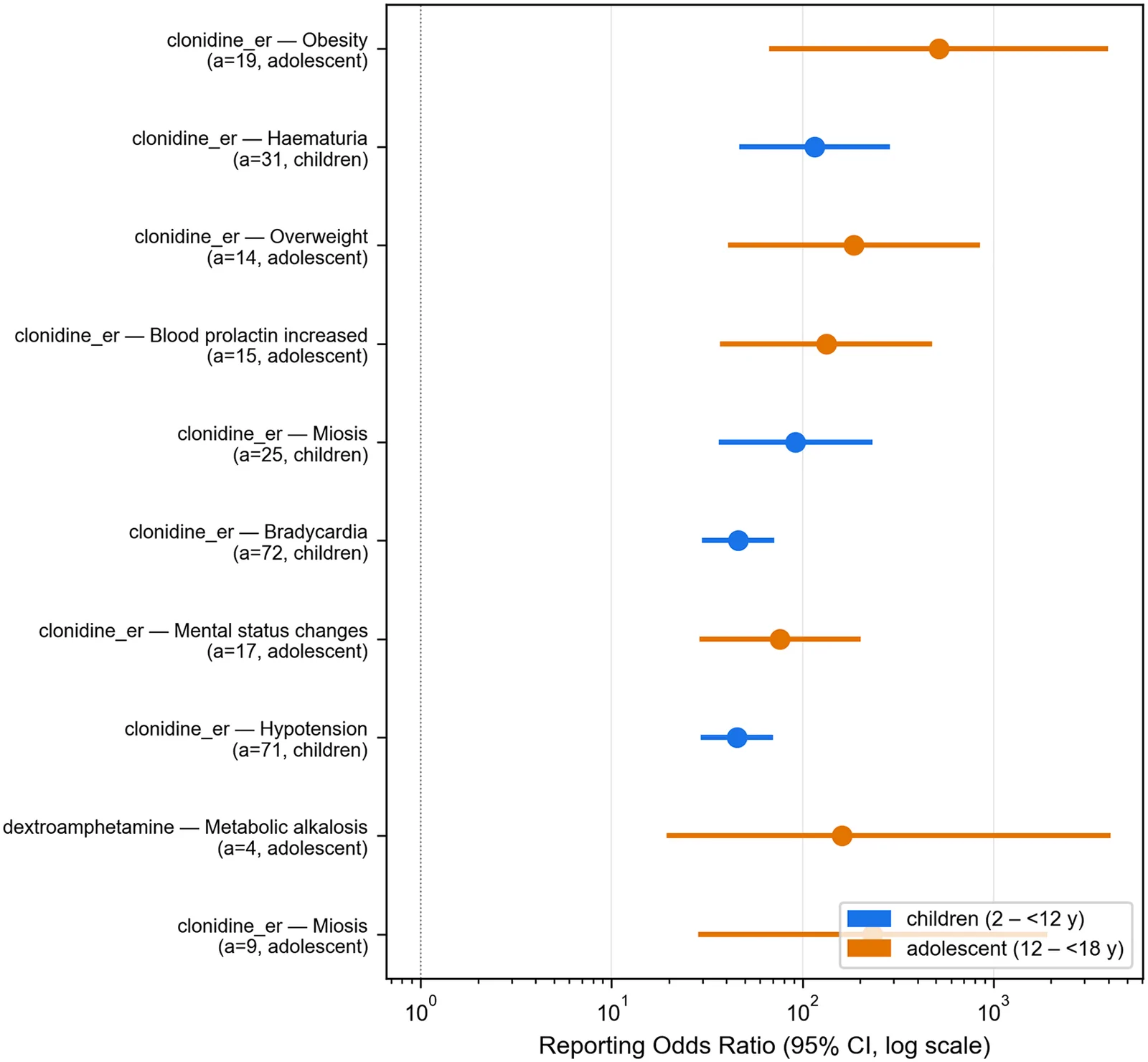
Forest plot of the top 10 strong signals ranked by IC_025_. Reporting odds ratios (point estimates, circles) with 95% confidence intervals (horizontal bars) for the ten highest-ranking Strong signals (four-method concordance) in the main analysis. Blue markers denote the children stratum (2 to <12 years); orange markers denote the adolescent stratum (12 to <18 years). *X*-axis is on a log10 scale; the vertical dotted line at ROR = 1 indicates the null. Each row is labeled by International Nonproprietary Name, MedDRA preferred term, observed case count (a), and age stratum. Signals with a < 5 used Mid-P exact confidence intervals in place of the log-normal approximation.

### A novel signal: dextroamphetamine and posterior reversible encephalopathy syndrome

A signal of particular clinical interest emerged for dextroamphetamine and posterior reversible encephalopathy syndrome (PRES) in adolescents. In the main analysis, this combination reached the Strong threshold (a = 19; ROR: 19.75, 95% CI: 11.19–34.86; PRR: 17.30, *χ*^2^ 192.96; IC_025_: 2.90; EBGM_05_: 7.67). Critically, this signal was reproduced as Strong in three analyses: the main within-class analysis, S3 (active-comparator restricted with methylphenidate as reference), and S6 (ICH E2B serious subset; a = 12, ROR: 17.10, 95% CI: 7.98–36.64, EBGM_05_: 4.08). Under the PS + SS relaxation (S1) it attenuated to a Moderate signal (3/4), missing the Strong threshold only on the conservative fallback-prior EBGM_05_ criterion while remaining positive on ROR, PRR and IC. To our knowledge, this represents the first systematic pharmacovigilance detection of a dextroamphetamine–PRES association at therapeutic doses. One prior published case report described PRES in an adolescent following Adderall XR overdose, but no prior FAERS study or FDA postmarketing review has reported this signal at therapeutic exposure (20).

### Sensitivity analyses

Results of the seven prespecified sensitivity analyses are summarized in Table 3. The intersection of main-analysis Strong signals with S1 (role_cod = PS + SS) and S6 (serious subset) yielded a consensus signal set of 29 signals detectable across all three analyses—these represent the most robust pharmacovigilance findings of the study. S1 and S6 were selected for this triangulation because they probe the two principal validity threats in within-class disproportionality from complementary directions: S1 (relaxing role_cod from primary suspect to primary-plus-secondary suspect) tests robustness to the drug-attribution assumption, whereas S6 (the ICH E2B serious-outcome subset) tests robustness to outcome severity and clinical relevance; signals concordant across the main analysis and both are therefore stable to both the attribution and the severity restriction.

**Table 3:** Summary of sensitivity analyses.

Analysis	Sample size	Strong signals	Key finding
**Main**	15,301	100	Reference
S1: role_cod = PS + SS	17,024	174	Adds 101 new signals; 73 of 100 main signals retained as Strong (27 attenuated under PS + SS); total 174 Strong
S2: Full pediatric FAERS comparator	15,301 (vs. 430,163 non-cases)	1,539 (3-method)	Methylphenidate remains with 0 Strong; main findings robust
S3: Active comparator (methylphenidate ref)	1,599	480	Non-methylphenidate signals markedly amplified
S4: New-drug branch (viloxazine, serdex)	335 (vs. 3,555 year-matched)	1	Immature signal landscape
S5: Infants-and-toddlers case series	296	— (descriptive)	Clonidine-ER dominant (41.9%); top PT “Foetal exposure during pregnancy”
S6: Serious outcomes (ICH E2B)	2,494	41	31 signals overlap with main; including dextroamphetamine–PRES
S7: Marketing era stratification	8,676/2,774/3,555	65/14/36	Signal detection highest in 2014–2017 era

ICH E2B, international council for Harmonisation E2B serious criteria; PRES, posterior reversible encephalopathy syndrome.

Of the ten signals in Table 2, five are members of the 29-signal consensus set (main ∩ S1 ∩ S6; Table 2, final column); the remaining five, although highly ranked by IC_025_, involve predominantly non-serious preferred terms that are sparsely represented in the ICH E2B serious subset (S6) and therefore do not meet the Strong threshold concordantly across all three analyses.

### Missing data patterns

Missingness for variables not entering the DA calculation — weight (64.7–69.2%), event date (39.4–54.4%), and derived sex field (8.9–13.7%) — was stratified by age group and reported in Figure 4. Age and reporter country were complete in the main analysis set by construction.

**Figure 4:**
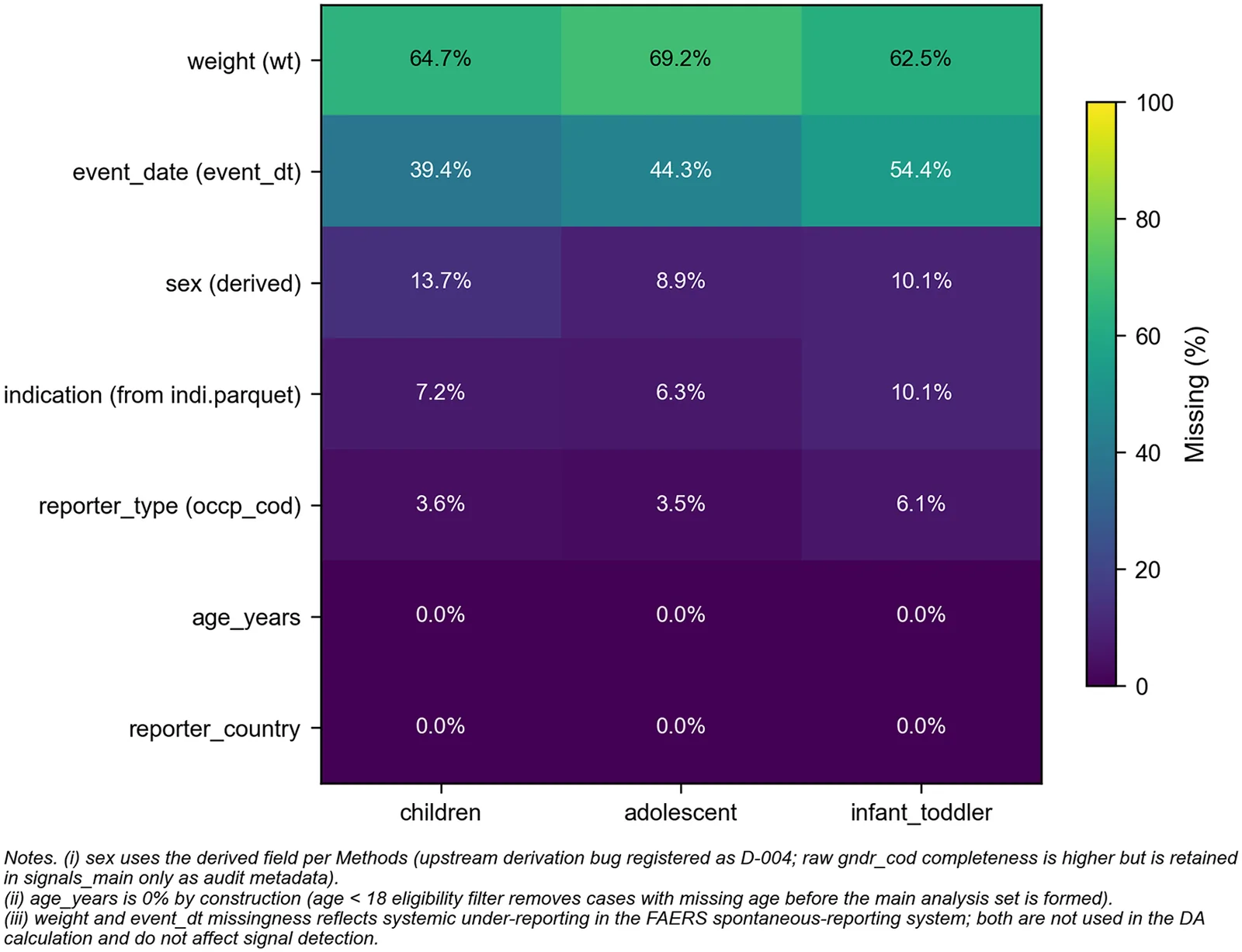
Missing/unknown rate per variable in the main analysis set, stratified by ICH age group. Percentage of records with missing or explicitly unknown values, across key demographic and reporting variables, stratified by age group. Rows are ordered by overall missingness (highest to lowest). (i) the sex field uses the derived encoding consistent with Methods; (ii) age_years shows 0% missingness by construction, as cases with missing age are excluded during pediatric (<18 years) eligibility filtering upstream of the main analysis set; (iii) weight (wt) and event_date (event_dt) missingness reflects systemic under-reporting inherent to FAERS spontaneous reports and does not enter the disproportionality calculation.

## Discussion

This retrospective disproportionality analysis of 15,301 unique pediatric attention-deficit/hyperactivity disorder (ADHD) reports in the United States Food and Drug Administration Adverse Event Reporting System (FAERS) from 2014 through 2024 identified 100 Strong drug–event signals (four-method concordance) under a within-class comparator design, stratified according to the International Council for Harmonisation E11(R1) developmental age framework. The most salient findings were (i) a marked concentration of Strong signals among α2-adrenergic agonists—clonidine extended-release (52 signals) and guanfacine extended-release (27)—which together accounted for 79% of all Strong signals; (ii) the conspicuous absence of Strong signals for methylphenidate and dexmethylphenidate despite their numerical dominance in the dataset, a pattern robust to both within-class and full pediatric FAERS comparator analyses; (iii) a pronounced adolescent-skewed distribution of signals (71 of 100 Strong signals in adolescents vs. 29 in children, representing a 3.8-fold per-capita enrichment); and (iv) a novel and clinically important signal linking dextroamphetamine to posterior reversible encephalopathy syndrome (PRES) in adolescents, reproducible across three independent sensitivity analyses.

The α2-agonist signal pattern is largely explicable on pharmacological grounds. Clonidine and guanfacine exert their therapeutic effect in ADHD through selective presynaptic α2A-adrenergic receptor agonism, resulting in reduced noradrenergic outflow from the locus coeruleus and enhanced prefrontal cortical function (21, 22). The same mechanism produces the well-characterized peripheral effects captured in our Strong signal list: bradycardia [Strong in children: reporting odds ratio (ROR) 46.0; 72 cases], hypotension (ROR 45.2; 71 cases), miosis (ROR 91.9; 25 cases), and mental status changes. These effects are consistent with product labeling and with prior pediatric pharmacovigilance literature (23). More unexpected, however, were signals for obesity and overweight in adolescent clonidine-ER users (Strong, ROR 516.0 and 185.6 respectively) and prolactin elevation. While sedation-associated reductions in energy expenditure and activity provide a plausible mechanistic bridge, the magnitude of disproportionality warrants formal prospective investigation, particularly given that α2-agonist prescribing in pediatric ADHD has expanded substantially following the 2020–2022 COVID-19–era increase in telepsychiatric ADHD diagnosis.

A hematuria signal for clonidine-ER in children (Strong, ROR 115.7; 31 cases) is not represented in current US Prescribing Information for either clonidine-ER or its immediate-release counterpart, nor in the guanfacine-ER label. Whether this reflects a class-wide α2-agonist effect, a specific clonidine-ER pharmaceutical attribute, or an ascertainment artifact driven by concomitant antihypertensive or psychotropic co-medications cannot be resolved by spontaneous-reporting data alone and represents a testable hypothesis for electronic health record–based pharmacoepidemiology.

The most clinically consequential finding of this study concerns the association between dextroamphetamine and PRES in adolescents. PRES is a neuroclinical syndrome characterized by subacute onset of headache, seizures, altered mental status, and visual disturbances, with the classical imaging finding of parieto-occipital vasogenic edema on magnetic resonance imaging (24, 25). The dominant pathophysiological mechanism is acute blood pressure elevation exceeding the limits of cerebral autoregulation, producing endothelial dysfunction and vasogenic edema (26). Dextroamphetamine, as a sympathomimetic agent that potently stimulates catecholamine release and inhibits reuptake, produces dose-dependent increases in systolic and diastolic blood pressure that are well documented in regulatory trials. The mechanistic linkage between amphetamine-induced hypertension and PRES is therefore biologically plausible, yet the signal has not been previously detected in large-scale pharmacovigilance analyses of ADHD medications. To our knowledge, only a single published case report, describing PRES in an adolescent following overdose of mixed amphetamine salts (Adderall XR), addresses this association (20). Importantly, our signal appears at therapeutic rather than supratherapeutic exposures and is reproducible in (i) the main within-class analysis, (ii) relaxation of the primary suspect constraint (S1), (iii) the active-comparator–restricted subset with methylphenidate as reference (S3), and (iv) restriction to ICH E2B–defined serious outcomes (S6). Neither the large FAERS ADHD medication analysis by Wu and colleagues (2025), nor the age-stratified analysis by Wei and colleagues (2023), nor the FDA's own post-marketing pharmacovigilance reviews of Mydayis and Dyanavel XR, have reported a PRES signal in this therapeutic class (9, 10, 27, 28). The identification of this previously unreported association, particularly one involving a serious neurological condition with potentially permanent sequelae in a minority of patients, should prompt targeted surveillance and case-series corroboration in pediatric neurology cohorts.

The absence of Strong signals for methylphenidate and dexmethylphenidate merits methodological commentary. Under the within-class comparator design, methylphenidate—representing 51.3% of the main analysis set—functions as the dominant component of the non-case reference pool against which signals for other agents are computed. This creates an inherent asymmetry: drug–event associations specific to methylphenidate itself are systematically attenuated because the comparator pool is saturated with methylphenidate exposures. We anticipated this and prespecified sensitivity analysis S2, which replaced the within-class comparator with the full pediatric FAERS pool (*n* = 430,163 non-cases). Methylphenidate remained without Strong signals even under this more discriminating comparator, indicating that its absence from the Strong signal list reflects a genuine absence of disproportionately reported adverse events within the pediatric ADHD context—not a methodological artifact. Further corroboration comes from sensitivity analysis S3, in which methylphenidate served as the explicit active comparator, yielding 480 Strong signals for the remaining seven active ingredients—a 4.8-fold amplification over the main analysis and strong evidence that other agents in the class carry signals that are masked when methylphenidate dominates the reference pool. These converging findings provide robust empirical grounding for methylphenidate's favorable comparative safety profile in pediatric ADHD, consistent with its position as first-line pharmacotherapy in major clinical guidelines (29).

The observed age-stratum asymmetry, with 3.8-fold enrichment of Strong signals in adolescents relative to children, merits careful interpretation. Several non-exclusive explanations may contribute. First, adolescents receive higher weight-adjusted doses on average and exhibit adult-like pharmacokinetics for most stimulants, yielding greater peak plasma concentrations and more pronounced pharmacodynamic effects. Second, adolescent ADHD pharmacotherapy is more likely to involve polypharmacy with psychotropic co-medications (antidepressants, antipsychotics, benzodiazepines), increasing the probability of drug–drug interactions that generate reportable adverse events; our data corroborate this (median concomitant drugs: adolescents 2 [1–3] vs. children 1 [1–2]). Third, adolescents are more likely to report adverse effects themselves, potentially capturing subjective symptoms (mood changes, somatic complaints) under-reported when children are the patients. Fourth, developmentally emerging comorbidities—affective disorders, substance use, eating disorders—may interact with stimulant pharmacology to produce age-specific signals not present in younger children. Disentangling these explanations requires patient-level longitudinal data beyond the spontaneous-reporting paradigm.

Our findings align with but meaningfully extend prior FAERS-based pediatric ADHD pharmacovigilance. Wu and colleagues (2025), analyzing 72,298 FAERS reports for methylphenidate, atomoxetine, and amphetamine over 2004–2023, identified psychiatric signals (psychosis, aggression) and cardiovascular signals (coronary and carotid artery dissection for amphetamines) but did not perform age stratification or include the α2-agonists and newer non-stimulants (9). Wei and colleagues (2023) introduced age stratification across 2014–2022 but, like Wu, included only three active ingredients, missed the α2-agonist signal concentration, and reported none of the findings we highlight here (10). Pozzi and colleagues (2019), examining mood- and emotion-related adverse drug reactions, confined their analysis to the pediatric stimulant–non-stimulant binary without drug-level or age-group granularity (30). Most recently, a real-world pharmacovigilance comparison of viloxazine and dextroamphetamine across all age groups has been reported using VigiAccess and FAERS (11), identifying broad differences in SOC-level event rates but without pediatric-specific age stratification, four-method concordance classification, or the PRES signal we detect here. The present study therefore represents the first pharmacovigilance analysis of pediatric ADHD medications to (i) include all eight currently FDA-approved first- and second-line agents (plus viloxazine and serdexmethylphenidate in a dedicated new-drug branch), (ii) apply the ICH E11(R1) developmental age framework to signal detection, (iii) combine four disproportionality methods with prespecified concordance thresholds, and (iv) report under the READUS-PV 2024 standard, with fully version-controlled, publicly archivable code.

Methodologically, several features distinguish the present analysis. Prespecification of the drug dictionary, age cutoffs, signal thresholds, and sensitivity analysis battery—locked in a version-controlled project document prior to data extraction—mitigates the *post-hoc* threshold tuning that has been identified as a methodological vulnerability in pharmacovigilance. The use of Mid-P exact confidence intervals for sparse cells (36.2% of tested combinations) addresses a well-recognized inferential weakness of asymptotic ROR and PRR estimates in low-count strata, an issue highlighted by recent critical reviews of disproportionality analysis (17). The independent cross-validation of ROR, PRR, and IC implementations against three literature-derived 2 × 2 ground-truth fixtures and against an independent statistical library provides an unusually strong internal consistency check (13–15).

A further consideration intrinsic to within-class disproportionality is masking (competition bias), whereby high-frequency drug–event associations inflate the expected counts and thereby attenuate the disproportionality of other associations sharing the same comparator pool (31). Because methylphenidate constitutes 51.3% of the within-class pool, its dominance could in principle mask signals for both methylphenidate itself and the co-analyzed agents. Two design features address this directly. First, sensitivity analysis S2 acts as a de-masking analysis: replacing the within-class comparator with the full pediatric FAERS pool (430,163 non-cases) removes methylphenidate's dominance of the reference, yet methylphenidate remained without any Strong signal—indicating that its null is not a masking artifact. Second, sensitivity analysis S3, using methylphenidate as the explicit active comparator, amplified signals for the remaining agents 4.8-fold, directly revealing associations that the within-class design partially attenuates. These analyses mitigate but do not wholly eliminate masking; consistent with the general observation that important masking is uncommon in large spontaneous-reporting databases and chiefly affects rarely reported events (32), we frame the within-class estimates as conservative and report the full pediatric comparator (S2) alongside them, while acknowledging that residual masking of low-frequency signals cannot be fully excluded.

Several limitations warrant explicit acknowledgment. First and most fundamentally, FAERS is a spontaneous-reporting system without denominator data on drug exposure; the disproportionality signals identified here are associations within the reporting population, not absolute incidence rates, and do not establish causality. Reporting biases, including the Weber effect (early post-marketing over-reporting), notoriety bias (publicized adverse effects over-reported), and channeling bias, are endemic to this data source (33). Second, 53.2% of reports in our dataset were consumer-initiated, a substantially higher proportion than classical pharmacovigilance databases dominated by healthcare-professional reporters; while this captures patient-experience data valuably, it also introduces variable reporting completeness and term-coding heterogeneity. Third, nearly half of cases (43.8%) carried indications other than or mixed with ADHD, suggesting substantial off-label prescribing and channeling effects that our within-class design only partially controls; sensitivity analysis restricted to ADHD-only indications was performed but included in supplementary reporting only. Fourth, the empirical Bayesian geometric mean for the adolescent stratum was computed under a fallback prior after automated hyperparameter fitting failed to converge; while this renders EBGM_05_ estimates conservative for this stratum, the direction and magnitude of signal enrichment were not affected. Fifth, the study time window (2014–2024) was selected to minimize MedDRA version drift at the expense of temporal breadth; signals potentially detectable in 2004–2013 but attenuated or amplified in recent years are beyond the present scope. Sixth, the infant-and-toddler stratum was necessarily descriptive rather than formally tested due to limited case counts (*n* = 296), although the striking 67.2% serious-event proportion within this stratum represents an independent pharmacovigilance flag deserving targeted investigation in pediatric intensive care and emergency medicine registries.

Beyond these specific limitations, broader interpretive caution is warranted. Disproportionality analysis identifies statistical patterns within a heavily biased reporting substrate, and the translation of such patterns into clinical recommendations requires corroboration through complementary evidence streams—cohort studies with exposure denominators, electronic health record–linked pharmacoepidemiology, and mechanistic pharmacological investigations. Three of the five pre-specified stratification variables (age group, drug class, and marketing era) were carried through into formal disproportionality comparisons; sex and reporter-country strata are descriptively presented in Table 1 but were not treated as independent signal-detection strata, to preserve statistical power in the ICH age categories of primary interest. We therefore frame all signals identified here as hypotheses warranting further investigation rather than as definitive safety concerns.

Future research priorities motivated by the present findings include: a dedicated FAERS re-analysis of the dextroamphetamine–PRES signal stratified by formulation and dose, coupled with systematic case-series collection from pediatric neurology referral centers; electronic health record–linked cohort studies of *α*2-agonist exposure and subsequent weight gain trajectories in adolescent ADHD; investigation of the clonidine-ER hematuria signal using renal biomarker–coded databases; and a meta-analytic synthesis of emerging pharmacovigilance signals for the 2021-approved viloxazine and serdexmethylphenidate as their post-marketing exposure accrues.

The clinical implications of these findings, while necessarily tentative given the methodological constraints enumerated, are nonetheless actionable. Clinicians prescribing dextroamphetamine to adolescents—particularly at doses associated with meaningful sympathomimetic load—may consider counseling patients and families about the atypical but serious PRES syndrome, may monitor blood pressure proactively, and may maintain a low threshold for neuroimaging in patients developing headache, visual disturbance, or altered mental status. Clinicians initiating α2-agonists in adolescents for ADHD may wish to counsel about potential weight changes, with consideration of metabolic monitoring in extended treatment. The unusually high serious-event proportion in the infant-and-toddler stratum provides empirical support for established guidance that ADHD pharmacotherapy should rarely if ever extend below the age-4 minimum endorsed by the American Academy of Pediatrics (29), and that any such use warrants specialist oversight.

## Conclusion

This age-stratified disproportionality analysis of 15,301 pediatric attention-deficit/hyperactivity disorder cases in the FDA Adverse Event Reporting System from 2014 through 2024 identified 100 Strong drug–event safety signals under prespecified four-method concordance criteria. Signals were disproportionately concentrated among α2-adrenergic agonists, which accounted for 79 percent of all Strong signals, and were approximately four-fold enriched in adolescents relative to children on a per-case basis. The study further identified a novel dextroamphetamine–posterior reversible encephalopathy syndrome association in adolescents, reproducible across three independent sensitivity analyses and not previously reported in large-scale pharmacovigilance investigations of ADHD medications. Methylphenidate, despite accounting for more than half the analytic dataset, yielded no Strong signals under either within-class or full pediatric FAERS comparator designs, providing empirical support for its established position as first-line pharmacotherapy. These findings, while hypothesis-generating and requiring confirmation through exposure-denominated pharmacoepidemiology, identify specific drug–event combinations warranting targeted surveillance, clinical monitoring, and mechanistic investigation.

## Data Availability

The original contributions presented in the study are included in the article/Supplementary Material, further inquiries can be directed to the corresponding author.
